# Vitamin D3 Regulates Follicular Development and Intrafollicular Vitamin D Biosynthesis and Signaling in the Primate Ovary

**DOI:** 10.3389/fphys.2018.01600

**Published:** 2018-11-14

**Authors:** Jing Xu, Maralee S. Lawson, Fuhua Xu, Yongrui Du, Olena Y. Tkachenko, Cecily V. Bishop, Lucas Pejovic-Nezhat, David B. Seifer, Jon D. Hennebold

**Affiliations:** ^1^Division of Reproductive and Developmental Sciences, Oregon National Primate Research Center, Oregon Health and Science University, Beaverton, OR, United States; ^2^Department of Obstetrics and Gynecology, School of Medicine, Oregon Health and Science University, Portland, OR, United States; ^3^Department of Reproductive Medicine, Tianjin Center Hospital of Gynecology Obstetrics, Tianjin, China; ^4^Department of Obstetrics, Gynecology and Reproductive Sciences, Yale School of Medicine, New Haven, CT, United States

**Keywords:** 25-hydroxyvitamin D3, 1α, 25-dihydroxyvitamin D3, vitamin D receptor, 1α-hydroxylase, 25-hydroxylase, rhesus macaque

## Abstract

There is an increasing recognition that vitamin D plays important roles in female reproduction. Recent studies demonstrated that 1α,25-dihydroxyvitamin D3 (VD3), the biologically active form of vitamin D, improved ovarian follicle survival and growth *in vitro*. Therefore, we investigated the direct effects of VD3 at the specific preantral and antral stages of follicular development, and tested the hypothesis that vitamin D receptor (VDR) and enzymes critical for vitamin D biosynthesis are expressed in the primate ovary. Fourteen adult rhesus macaques provided ovarian tissue. Secondary and antral follicles were isolated for PCR analysis on VDR, vitamin D3 25-hydroxylase, and 25-hydroxyvitamin D3-1α-hydroxylase. VDR protein localization was determined by immunohistochemistry on ovarian sections. Isolated secondary follicles were cultured under conditions of control and VD3 supplementation during the preantral or antral stage. Follicle survival, growth, steroid and anti-Müllerian hormone (AMH) production, as well as oocyte maturation were evaluated. *In vivo*- and *in vitro*-developed follicles were also assessed for genes that are critical for vitamin D biosynthesis and signaling, gonadotropin signaling, steroid and paracrine factor production, and oocyte quality. The mRNA encoding VDR, 25-hydroxylase, and 1α-hydroxylase was detectable in *in vivo*- and *in vitro*-developed preantral and antral follicles. The 25-hydroxylase was elevated in cultured follicles relative to *in vivo*-developed follicles, which further increased following VD3 exposure. VD3 treatment increased 1α-hydroxylase in *in vitro*-developed antral follicles. The absence of VD3 during culture decreased VDR expression in *in vitro*-developed antral follicles, which was restored to levels comparable to those of *in vivo*-developed antral follicles by VD3 supplementation. Positive immunostaining for VDR was detected in the nucleus and cytoplasm of granulosa cells and oocytes. While only survival was improved in preantral follicles treated with VD3, VD3 supplementation promoted both survival and growth of antral follicles with increased estradiol and AMH production, as well as oocyte maturation. Thus, Vitamin D biosynthesis and signaling systems are expressed in primate ovarian follicles. Our findings support a role for VD3 in regulating follicular development in a stage-dependent manner, as well as the intrafollicular vitamin D biosynthesis and signaling, directly in the ovary.

## Introduction

Vitamin D3 (cholecalciferol) is produced in the skin or obtained from diet. Vitamin D3 25-hydroxylase encoded by cytochrome P450 family 2 subfamily R polypeptide 1 (*CYP2R1*), converts vitamin D3 to 25-hydroxyvitamin D3 (calcifediol). Through actions of 25-hydroxyvitamin D3-1α-hydroxylase, encoded by cytochrome P450 family 27 subfamily B polypeptide 1 (*CYP27B1*), 25-hydroxyvitamin D3 is converted to 1α,25-dihydroxyvitamin D3 (VD3; calcitriol) (Bikle, 2014). Although the kidney is the primary source of circulating VD3, other organs, including the ovary, also express 1α-hydroxylase, which could catalyze VD3 biosynthesis (Bikle, 2014; Xu et al., 2016c) (RNA-Seq of macaca mulatta: adult female ovarian follicle^1^; accession number: SRP044327). Therefore, the regulation of ovarian function by vitamin D may be mediated via its endocrine and paracrine actions. VD3 regulates cellular function through vitamin D receptor (VDR) (Bikle, 2014). VDR is expressed in various organs, including the hypothalamus, pituitary, ovary, oviduct, uterus and placenta (Stumpf and Denny, 1989; Xu et al., 2016c) (RNA-Seq of macaca mulatta: adult female ovarian follicle;^1^ accession number: SRP044327), which could alter their function through VDR activation by circulating or locally synthesized VD3. Thus, ovarian parameters need to be evaluated in the presence of physiological levels of VD3, especially under non-pathological conditions, to assess the direct impact of VD3 on processes critical for the development of follicles yielding oocytes that undergo fertilization and subsequent embryonic development.

The effect of vitamin D on ovarian function was studied primarily in the clinical context of its impact on obstetrical outcomes, particularly in patients with ovarian disorders seeking fertility treatment (Irani and Merhi, 2014; Muscogiuri et al., 2017). To date, data are not consistent regarding the relationship between follicular fluid vitamin D concentrations and pregnancy rates, with positive (Ozkan et al., 2010), negative (Anifandis et al., 2010), and insignificant (Firouzabadi et al., 2014) correlations reported. However, vitamin D supplementation appears to be beneficial to women suffering from obesity and insulin resistance. Studies in patients with polycystic ovary syndrome indicated that vitamin D supplementation improved follicular growth, dominant follicle formation, and pregnancy rates, as well as supported the resumption and maintenance of menstrual cycles (Thys-Jacobs et al., 1999; Fang et al., 2017). A multi-center study suggested that vitamin D deficiency in late-reproductive-age women (>40 years) correlated with a greater decrease in ovarian reserve (Merhi et al., 2012). However, mechanisms through which vitamin D regulates follicular function and oocyte quality remain obscure due to the lack of adequate research models.

Previous animal studies indicated that female mice maintained on a vitamin D-deficient diet after puberty had arrested follicular development and prolonged estrous cycles, with less oocytes retrieved from oviducts following gonadotropin stimulation (Dicken et al., 2012). VDR null female mice exhibited ovarian insufficiencies with impaired follicular development (Kinuta et al., 2000). Estrogen biosynthesis was disrupted due to the decreased gene expression and activity of aromatase. It appears that vitamin D is essential for proper ovarian function and maintenance of female reproductive physiology. In order to rule out systemic effects caused by global manipulations, e.g., elevated serum luteinizing hormone (LH) and follicle-stimulating hormone (FSH) in VDR mutant mice (Kinuta et al., 2000), we utilized follicle culture approach allowing direct vitamin D treatment of follicles developed *in vitro* (Xu et al., 2016b). Our data revealed that VD3 supplementation improved macaque follicle survival, growth and anti-Müllerian hormone (AMH) production *in vitro*, though oocyte competence was not assessed.

In the present study, instead of covering the entire follicle culture period, vitamin D was supplemented during the specific stages of follicular development to examine the direct actions of VD3 on preantral and antral follicle growth and function, including steroid secretion and oocyte maturation. Moreover, experiments were conducted to test the hypothesis that VDR and enzymes critical for vitamin D biosynthesis are expressed in follicles developed *in vivo* and *in vitro*. Endpoints also included VD3 effects on expression of genes that are critical for gonadotropin signaling, steroid and paracrine factor production, as well as oocyte quality.

## Materials and Methods

### Animals and Ovary Collection

The general care and housing of rhesus macaques (*Macaca mulatta*) were provided by the Division of Comparative Medicine, Oregon National Primate Research Center (ONPRC), Oregon Health and Science University (OHSU). Diet consisted of Purina monkey chow containing stabilized VD3 (6.6 IU/g; Ralston-Purina, Richmond, IN, United States). This study was carried out in accordance with principles of the National Institutes of Health’s Guide for the Care and Use of Laboratory Animals. The protocol was approved by the ONPRC Institutional Animal Care and Use Committee (Xu et al., 2016b).

A total of 14 adult female macaques (6–14 years old) provided ovarian tissue. Six animals exhibiting regular menstrual cycles were assigned to the study. Ovariectomies were conducted at the early follicular phase (cycle day 1–4). Ovaries from the additional 8 animals were collected through the ONPRC Pathology Services Unit tissue distribution program. Euthanasia was due to reasons unrelated to reproductive health, e.g., chronic colitis and reactive arthritis. Ovaries were transferred into HEPES-buffered holding media (Cooper Surgical, Inc., Trumbull, CT) at 37^o^C (Xu et al., 2016a).

### Presence of Vitamin D Biosynthesis and Signaling Components in the Ovary

Half of one ovary from the euthanized animals (*n* = 8) was used to assess the presence of components involved in vitamin D biosynthesis and signaling in preantral and antral follicles. As described previously (Xu et al., 2016c), the ovarian cortex was cut into 0.5 × 0.5 × 0.5 mm cubes for secondary follicle (diameter = 125–225 μm) isolation, while small antral follicles (diameter = 0.5–1.5 mm) were isolated from the medulla. Follicles from 2 animals were pooled to generate 4 pools from 8 animals (30 secondary or 10 antral follicles/pool), and transferred to the lysis buffer of an Absolutely RNA Nanoprep Kit (Agilent Technologies, Santa Clara, CA, United States) for RNA isolation. RNA was reverse-transcribed into cDNA using a GoScript Reverse Transcription System (Promega Corporation, Madison, WI, United States) (Xu et al., 2016c). PCR primers were designed using NCBI/Primer-BLAST (National Institutes of Health, Bethesda, MD, United States) for *CYP2R1*, *CYP27B1*, and *VDR* (Table 1). Qualitative PCR was conducted using GoTaq Green Master Mix (Promega Corporation) on the Eppendorf Mastercycler Nexus GX2 (Eppendorf, Hauppauge, NY): 95°C/1 min followed by 36 cycles of 95°C/30 s, 58°C/45 s, and 68°C/45 s. The final extension was at 72°C for 3 min. PCR products were purified using a QIAquick PCR Purification Kit (QIAGEN Inc., Valencia, CA, United States) and sequenced using a 3730xl DNA Analyzer (Thermo Fisher Scientific, Waltham, MA, United States) by the ONPRC Molecular and Cellular Biology Core to verify their identity (Xu et al., 2005).

**Table 1:** PCR primers and real-time PCR Assay IDs.

Gene	PCR primers and real-time PCR Assay IDs	
**PCR primers**	**Forward**	**Reverse**
*CYP2R1^a^*	TGGGGCAGAGGAAAAACTGA	AGACTAACACAAAGGCGGGT
*CYP27B1*	GACTGCTCACTGCGGAAGG	GGAACAGGAAGTGGGTCAGG
*VDR*	GTCCGTGCTCCGCTTTAGAT	GTAGGTGGGGTCGTAGGTCT
**Real-Time PCR Assay IDS**		
*AMH*	Mm00431795_g1	
*AMHR2*	Mm00513847_m1	
*BMP15*	Hs00193764_m1	
*CYP17A1*	Hs01124136_m1	
*CYP19A1*	Hs00903413_m1	
*CYP27B1*	Hs01096154_m1	
*CYP2R1*	Hs01379776_m1	
*FSHR*	Rh01026045_m1	
*GDF9*	Hs03986126_s1	
*LHCGR*	Hs00174885_m1	
*VDR*	Rh02828247_m1	

^a^*AMH, anti-Müllerian hormone; AMHR2, AMH receptor II; BMP15, bone morphogenetic protein 15; CYP17A1, cytochrome P450 family 17 subfamily A polypeptide 1; CYP19A1, cytochrome P450 family 19 subfamily A polypeptide 1; CYP27B1, cytochrome P450 family 27 subfamily B polypeptide 1; CYP2R1, cytochrome P450 family 2 subfamily R polypeptide 1; FSHR, FSH receptor; GDF9, growth differentiation factor 9; LHCGR, luteinizing hormone/choriogonadotropin receptor; VDR, vitamin D receptor.*

To identify follicular cells responsive to VD3, VDR protein localization was determined by immunohistochemistry. The remaining half of the ovary from euthanized animals (*n* = 8) was fixed and embedded in paraffin by the ONPRC Histopathology-Morphology Research Core. Deparaffinized 5 μm sections were rehydrated in PBS followed by incubation at 4°C overnight with mouse anti-human VDR antibody (1:50; sc-13133; Santa Cruz Biotechnology, Inc., Santa Cruz, CA, United States). Mouse non-immune IgG was used as the negative control. Sections were then incubated with the secondary antibody and processed using a VECTASTAIN Elite ABC Kit (Vector Laboratories, Inc., Burlingame, CA; PK-6102 biotinylated anti-mouse IgG). The antigen-antibody complex was visualized by incubation with 3,3’-diaminobenzidine. Select sections were counterstained using hematoxylin to demonstrate the nuclear versus cytoplasmic staining of VDR. Images were captured via an Olympus BX40 inverted microscope and an Olympus DP72 digital camera (Olympus Imaging America Inc., Center Valley, PA, United States) (Xu et al., 2016c).

### VD3 Regulation of Gene Expression in Preantral and Antral Follicles

This experiment contained the control and VD3 supplementation groups with four biological replicates in each group. Secondary and small antral follicles were isolated from the second ovary of euthanized animals (*n* = 8), as described above. Antral follicles from 2 animals were pooled to generate four pools from 8 animals (10 follicles/pool), for RNA isolation and reverse-transcription, as described above. These samples represent *in vivo*-developed antral follicles. Secondary follicles were cultured individually, as reported previously (Xu et al., 2016b). Briefly, follicles were transferred into 5 μl 0.15% (w/v) sodium alginate (FMC Biopolymer AS d/b/a NovaMatrix, Sandvika Norway)-PBS, which were then encapsulated in 50 mM CaCl_2_, 140 mM NaCl, 10 mM HEPES solution (pH 7.2). Encapsulated follicles were cultured at 37°C and 5% O_2_ (in 6% CO_2_/89% N_2_) in 300 μl α-minimum essential medium (Thermo Fisher Scientific, Waltham, MA, United States) containing 6% (v/v) human serum protein supplement (Cooper Surgical, Inc.), 1 ng/ml FSH (NV Organon/Merck Sharp & Dohme, Oss, Netherlands), 5 μg/ml insulin, 5 μg/ml transferrin, 5 ng/ml sodium selenite, 0.5 mg/ml bovine fetuin, and 10 μg/ml gentamicin (Sigma-Aldrich, St Louis, MO, United States). Follicles from each animal were randomly assigned to two experiments with two groups in each experiment (12 follicles/animal/group): Experiment 1 – (a) 0.025% ethanol vehicle control and (b) 25 pg/ml VD3 (biologically active form; Sigma-Aldrich) (Xu et al., 2016b) supplementation during weeks 0–2 (preantral stage); Experiment 2 – (a) control and (b) 25 pg/ml VD3 supplementation during weeks 3–5 (antral stage). Media (150 μl) was replaced every other day. Follicle survival and antrum formation were assessed weekly using an Olympus CK-40 inverted microscope and an Olympus DP11 digital camera (Olympus Imaging America Inc., Center Valley, PA, United States), as described previously (Xu et al., 2016b). Follicles were considered atretic if the oocyte was dark or not surrounded by a layer of granulosa cells, the granulosa cells appeared dark or fragmented, or the follicle diameter decreased. For Experiment 1, surviving preantral follicles were harvested at the end of week 2. For Experiment 2, surviving small antral follicles were harvested at the end of week 5. Follicles from 2 animals were pooled to generate 4 pools from 8 animals (15 preantral or 10 antral follicles/pool/group), for RNA isolation and reverse-transcription, as described above. These samples represent *in vitro*-developed preantral and antral follicles.

Quantitative real-time PCR was performed for *in vivo*- and *in vitro*-developed follicles using the TaqMan Gene Expression Assays and Applied Biosystems 7900HT Fast Real-time PCR System (Thermo Fisher Scientific, Waltham, MA, United States) (Xu et al., 2016c) for genes that are responsible for vitamin D biosynthesis and signaling (*CYP2R1*, *CYP27B1*, and *VDR*), gonadotropin signaling (FSH receptor, *FSHR*; LH/choriogonadotropin receptor, *LHCGR*), steroid and paracrine factor production and signaling (cytochrome P450 family 17 subfamily A polypeptide 1, *CYP17A1*; cytochrome P450 family 19 subfamily A polypeptide 1, *CYP19A1*; *AMH*; AMH receptor II, *AMHR2)*, as well as oocyte quality (bone morphogenetic protein 15, *BMP15*; growth differentiation factor 9, *GDF9*) (Table 1). Mitochondrial ribosomal protein S10 (*MRPS10*) served as the internal control (Bishop et al., 2012). *In vitro*-developed follicles with and without VD3 treatment were compared to examine VD3 actions on follicular gene expression. Antral follicles developed *in vitro* and *in vivo* were also compared to assess effects of *in vitro* manipulation on expression of genes that are responsible for vitamin D biosynthesis and signaling.

### Direct Actions of Vitamin D on Preantral and Antral Follicle Development *in vitro*

This experiment contained the control, preantral VD3 supplementation, and antral VD3 supplementation groups with six animals in each group. Secondary follicles were isolated from ovaries of assigned animal (*n* = 6), encapsulated in alginate, and cultured, as described above. Follicles from each animal were randomly assigned to three groups (12 follicles/animal/group): (a) control; (b) 25 pg/ml VD3 supplementation during weeks 0–2; and (c) 25 pg/ml VD3 supplementation during weeks 3–5. Follicle survival and antrum formation were assessed, as described above. Follicle survival was presented as percentages of surviving follicles versus total follicles cultured. Follicle photographs were imported into ImageJ 1.50 software (National Institutes of Health), and the diameter of each follicle was measured, as described previously (Xu et al., 2016b).

Media was collected and replaced every other day, and stored at -20°C. Media samples were pooled by week for each follicle, animal and group for analyses of steroid and paracrine factor concentrations by the ONPRC Endocrine Technologies Core. Progesterone (P4) and estradiol (E2) were assayed using a Cobas Elecsys platform (Roche Diagnostics, Indianapolis, IN, United States). The detection ranges were 0.05–60.0 ng/ml and 5–3000 pg/ml for P4 and E2, respectively. ELISAs were performed to measure androstenedione using an AA E-1000 kit (Rocky Mountain Diagnostics, Inc., Colorado Springs, CO, United States) (Xu et al., 2016b), and AMH using an AL-105 kit (AnshLabs, Webster, TX, United States) (Xu et al., 2016b).

At the end of week 5, *in vitro*-developed antral follicles were treated with 100 ng/ml recombinant human chorionic gonadotropin (hCG; Merck Serono, Geneva, Switzerland) for 34 h. Cumulus-oocyte complexes were collected by dissecting the follicle wall in the Tyrode’s albumin lactate pyruvate (TALP)-HEPES-BSA (0.3% v/v) media. Denuded oocytes were assessed for diameters and meiotic status, as previously described (Xu et al., 2016a).

### Statistical Analysis

Statistical significance was determined by SigmaPlot 11 software (SPSS, Inc., Chicago, IL, United States). Data involving three groups were analyzed using a one-way ANOVA followed by the Student-Newman-Keuls *post hoc* test. Data involving two groups were analyzed using a Student’s *t*-test. The non-parametric one-way ANOVA was used to evaluate follicle survival (Wilcoxon Scores), with six animals in each group. Exact *P*-values (chi-square) were determined by the Kruskal-Wallis Test. The mRNA levels represent data from 4 biological replicates pooled from 8 animals in each group. Follicle and oocyte diameters, as well as steroid and AMH concentrations, were analyzed for each follicle with total follicle numbers indicated in the figure legends, and represent follicles obtained from six animals. Differences were considered significant at *P* < 0.05 and values are presented as mean ± SEM.

## Results

### Follicular Vitamin D Biosynthesis Enzyme and VDR Expression *in vivo* and *in vitro*

The mRNA encoding *CYP2R1*, *CYP27B1*, and *VDR* was present in all pooled samples of secondary and small antral follicles isolated from ovaries of 8 macaques (data not shown). Amplicon identities were confirmed by sequencing the PCR products.

**FIGURE 1:**
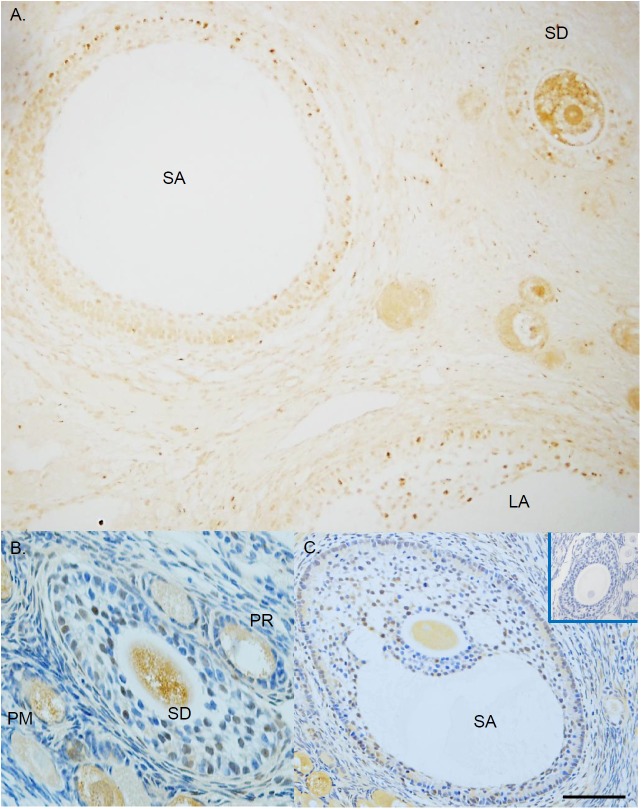
The expression of vitamin D receptor (VDR) in rhesus macaque follicles developed *in vivo*. Immunohistochemistry was performed without **(A)** or with **(B,C)** counterstain hematoxylin to allow for visualization of the VDR nuclear and cytoplasmic staining. Mouse non-immune IgG was used as the negative control (**C** insert). PM, primordial follicle; PR, primary follicle; SD, secondary follicle; SA, small antral follicle; LA, large antral follicle. Scale bar = 100 μm for **(A,C)**, and 50 μm for **(B)**.

Positive immunostaining for VDR was detected in the nucleus and cytoplasm of granulosa cells and oocytes in follicles (brown; Figure 1A). VDR staining was not evident in pre-granulosa cells of the primordial follicles (PM; Figure 1B), and was minimal in granulosa cells of the primary follicles (PR; Figure 1B). Positive staining in granulosa cells became apparent as follicles progressed to the secondary (SD; Figures 1A,B), small antral (SA; Figures 1A,C), and large antral (LA; Figure 1A) stages. VDR staining was present in the oocyte of follicles from all developmental stages (Figures 1A–C). Immunostaining for VDR was absent in the negative control sections (Figure 1C, insert).

**FIGURE 2:**
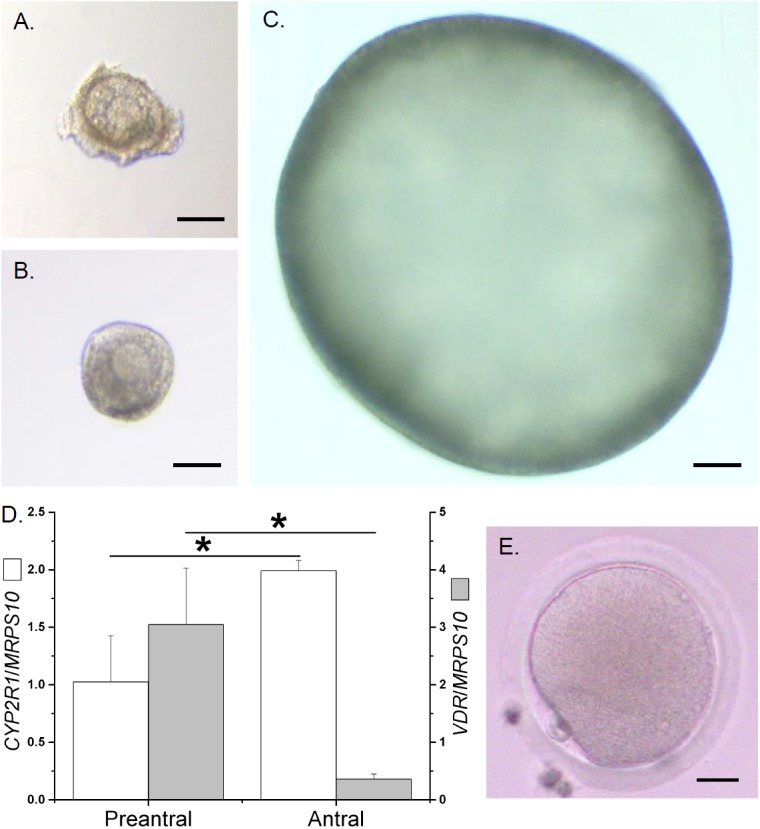
The growth, as well as mRNA levels of cytochrome P450 family 2 subfamily R polypeptide 1 (*CYP2R1*) and vitamin D receptor (*VDR*), of rhesus macaque follicles developed *in vitro*. A representative isolated secondary follicle **(A)** survived the first 2 weeks of culture under control conditions **(B)**, and then grew to the small antral stage at week 5 **(C)**. The mRNA levels of *CYP2R1* and *VDR* in preantral (week 2) and antral (week 5) follicles developed *in vitro* under control conditions were determined by real-time PCR (4 pools from 8 animals, with follicles from 2 animals per pool) **(D)**. Mitochondrial ribosomal protein S10 (*MRPS10*) served as the internal control. Following recombinant human chorionic gonadotropin exposure, a metaphase II oocyte was harvested from *in vitro*-developed antral follicles treated with 1α,25-dihydroxyvitamin D3 during culture weeks 3–5 **(E)**. ^∗^Significant difference between preantral and antral follicles, *P* < 0.05. Data are presented as the mean ± SEM. Scale bar = 100 μm for follicles and 25 μm for the oocyte.

Sixty percent of secondary follicles (Figure 2A) survived the first 2 weeks of culture under control conditions, possessing an intact basement membrane, multiple granulosa layers, and a healthy centrally-located oocyte (Figure 2B). While 57% of the surviving follicles remained at the preantral stage, 43% formed an antrum at week 3 and grew to the small antral stage at week 5 (Figure 2C). The mRNA expression of *CYP2R1*, *CYP27B1*, and *VDR* were detectable in *in vitro*-developed preantral follicles at week 2 and small antral follicles at week 5. While the mRNA levels of *CYP27B1* were not different between preantral and small antral follicles developed *in vitro* (*CYP27B1*/*MRPS10* = 0.01 ± 0.01 versus 0.03 ± 0.01), the mRNA levels of *CYP2R1* were higher (*P* < 0.05), whereas the mRNA levels of *VDR* were lower (*P* < 0.05), in small antral follicles relative to those of preantral follicles (Figure 2D).

### VD3 Actions in Preantral Follicles

**FIGURE 3:**
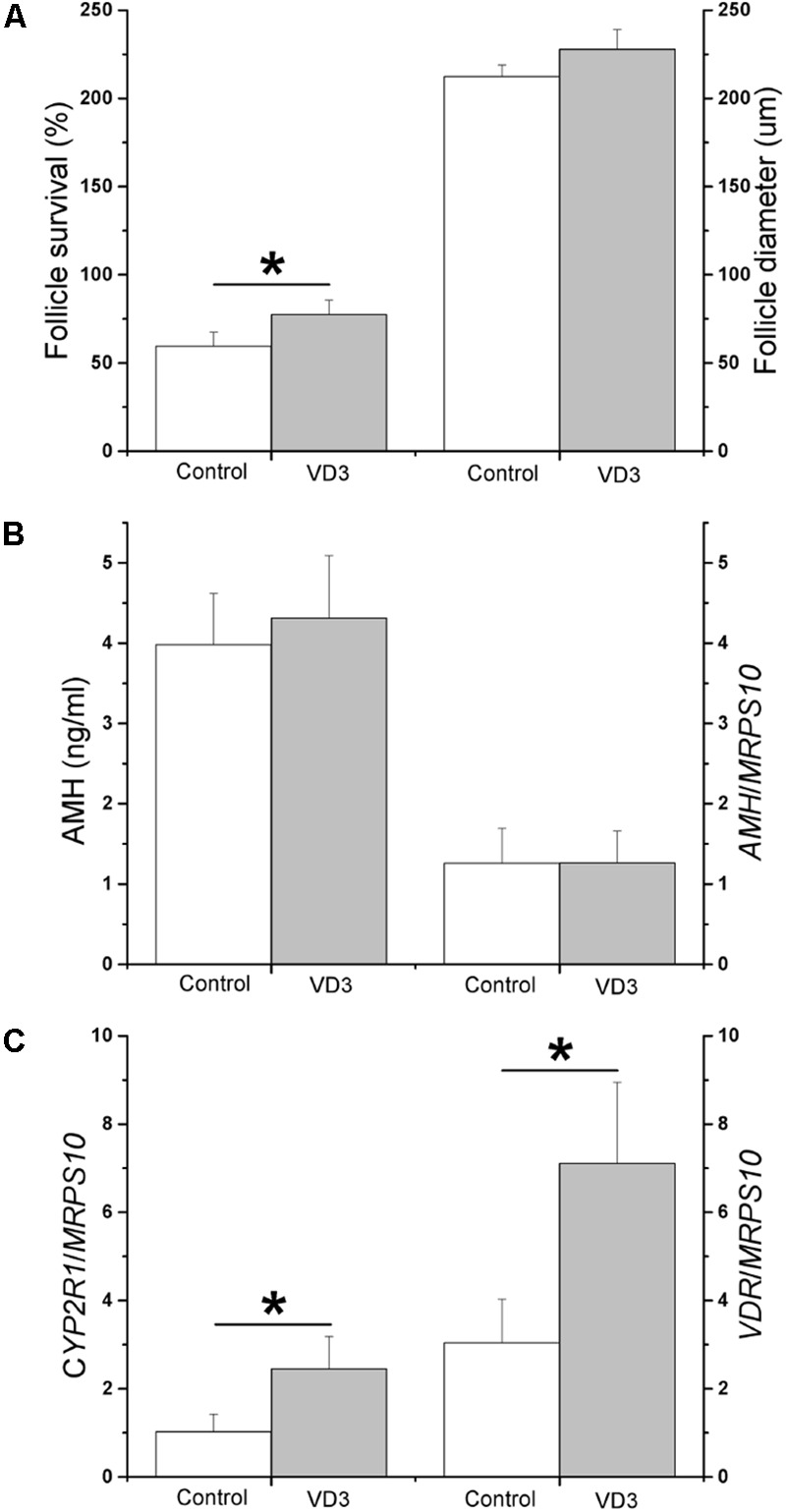
Effects of 1α,25-dihydroxyvitamin D3 (VD3) supplementation during weeks 0–2 (preantral stage) on the development and function of rhesus macaque preantral follicles developed *in vitro*. Follicle survival was calculated as the percentage of surviving preantral follicles relative to the total follicles cultured (*n* = 6 animals per group) **(A)**. Follicle growth was determined by measuring follicle diameters (16–17 follicles assessed per group) **(A)**. Anti-Müllerian hormone (AMH) concentrations in the culture media were measured by ELISA (16–17 follicles assessed per group) **(B)**. *AMH* mRNA levels in cultured follicles were determined by real-time PCR (4 pools from 8 animals, with follicles from 2 animals per pool) **(B)**. The mRNA levels of cytochrome P450 family 2 subfamily R polypeptide 1 (*CYP2R1*) and vitamin D receptor (*VDR*) were determined by real-time PCR (4 pools from 8 animals, with follicles from 2 animals per pool) **(C)**. Mitochondrial ribosomal protein S10 (*MRPS10*) served as the internal control. ^∗^Significant difference between experimental groups, *P* < 0.05. Data are presented as the mean ± SEM.

With VD3 exposure during the first 2 weeks (preantral stage), preantral follicle survival increased (*P* < 0.05) at the end of week 2 (Figure 3A; presented as percentages of surviving preantral follicles/total follicles cultured). At week 2, preantral follicle diameters (Figure 3A) and media AMH concentrations (Figure 3B) were not altered following VD3 treatment. While mRNA levels of *CYP27B1* were comparable between the control and VD3-treated preantral follicles (*CYP27B1*/*MRPS10* = 0.01 ± 0.01 versus 0.1 ± 0.1), mRNA levels of *CYP2R1* and *VDR* increased (*P* < 0.05) in preantral follicles treated with VD3 (Figure 3C). At the end of week 2, preantral follicles from the VD3-treated group were comparable with those from the control group in terms of mRNA levels of *AMH* (Figure 3B), *AMHR2*, *FSHR*, *BMP15*, and *GDF9* (Table 2).

**Table 2:** Gene expression in cultured macaque follicles.

Gene	Control^a^	VD3
**Preantral follicle**		
*AMHR2^b^*	1.3 ± 0.2	1.3 ± 0.2
*FSHR*	1.9 ± 0.4	3.0 ± 0.8
*BMP15*	3.5 ± 0.9	7.2 ± 2.7
*GDF9*	2.9 ± 0.7	4.6 ± 1.3
**Antral follicle**		
*AMHR2*	0.9 ± 0.1	0.8 ± 0.1
*LHCGR*	2.8 ± 0.8	4.0 ± 0.9
*CYP17A1*	0.7 ± 0.3	0.9 ± 0.5
*CYP19A1*	7.6 ± 1.5	11.3 ± 2.6

^a^*Data represent 4 pooled samples from 8 animals, with follicles from 2 animals per sample, in each experimental group. Values represent the relative expression of the target gene versus the expression of invariant mitochondrial ribosomal protein S10, and are the mean ± SEM with each sample as an individual data point. ^*b*^VD3, 1α,25-dihydroxyvitamin D3 supplementation; AMHR2, anti-Müllerian hormone receptor II; BMP15, bone morphogenetic protein 15; CYP17A1, cytochrome P450 family 17 subfamily A polypeptide 1; CYP19A1, cytochrome P450 family 19 subfamily A polypeptide 1; FSHR, FSH receptor; GDF9, growth differentiation factor 9; LHCGR, luteinizing hormone/choriogonadotropin receptor.*

### VD3 Actions in Antral Follicles

**FIGURE 4:**
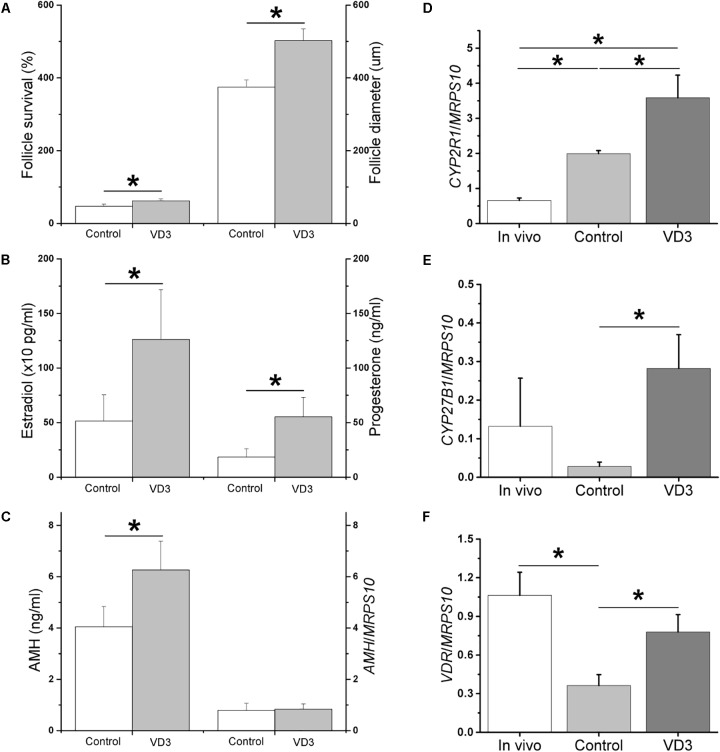
Effects of 1α,25-dihydroxyvitamin D3 (VD3) supplementation during weeks 3–5 (antral stage) on the development and function of rhesus macaque antral follicles developed *in vitro*. Follicle survival was calculated as the percentage of surviving antral follicles relative to the total follicles cultured (*n* = 6 animals per group) **(A)**. Follicle growth was determined by measuring follicle diameters (12–19 follicles assessed per group) **(A)**. Estradiol and progesterone concentrations in the culture media were measured by the electrochemiluminescence immunoassay (12–19 follicles assessed per group) **(B)**. Anti-Müllerian hormone (AMH) concentrations in the culture media were measured by ELISA (12–19 follicles assessed per group) **(C)**. *AMH* mRNA levels in cultured follicles were determined by real-time PCR (4 pools from 8 animals, with follicles from 2 animals per pool) **(C)**. The mRNA levels of cytochrome P450 family 2 subfamily R polypeptide 1 (*CYP2R1*) **(D)**, cytochrome P450 family 27 subfamily B polypeptide 1 (*CYP27B1*) **(E)**, and vitamin D receptor (*VDR*) **(F)** were determined by real-time PCR (4 pools from 8 animals, with follicles from 2 animals per pool). Mitochondrial ribosomal protein S10 (*MRPS10*) served as the internal control. ^∗^Significant difference between experimental groups, *P* < 0.05. Data are presented as the mean ± SEM.

VD3 exposure during weeks 3–5 (antral stage) increased (*P* < 0.05) antral follicle survival at the end of week 5 (Figure 4A; presented as percentages of surviving antral follicles/total follicles cultured). Antral follicle diameters were larger (*P* < 0.05) following VD3 treatment (Figure 4A). At week 5, while media androstenedione concentrations were comparable between the control and VD3-treated groups (12 ± 4 pg/ml versus 20 ± 5 pg/ml), media concentrations of E2, P4 (Figure 4B) and AMH (Figure 4C) increased (*P* < 0.05) in the VD3-treated group. The mRNA levels of *CYP2R1* were higher (*P* < 0.05) in *in vitro*-developed, compared with those of *in vivo*-developed, small antral follicles, which further increased (*P* < 0.05) following VD3 treatment (Figure 4D). There were no differences in mRNA levels of *CYP27B1* between *in vivo*- and *in vitro*-developed small antral follicles, though VD3 treatment increased (*P* < 0.05) *CYP27B1* mRNA levels in cultured follicles relative to those of controls (Figure 4E). The mRNA levels of *VDR* were lower (*P* < 0.05) in *in vitro*-developed, compared with those of *in vivo*-developed, small antral follicles, which increased (*P* < 0.05) following VD3 treatment to levels observed in small antral follicles developed *in vivo* (Figure 4F). At the end of week 5, antral follicles from the VD3-treated group were comparable with those from the control group in terms of mRNA levels of *AMH* (Figure 4C), *AMHR2*, *LHCGR*, *CYP17A1*, and *CYP19A1* (Table 2). Following hCG treatment, the percentages of healthy oocytes harvested from the control and VD3-treated antral follicles were approximately 65 and 77%, respectively (Table 3). While all healthy oocytes from the control group remained at the germinal vesicle intact (GV) stage, 2 oocytes from the VD3-treated group resumed meiosis, wherein one progressed to the metaphase I (MI; diameter = 108 μm) and the other to the metaphase II (MII; diameter = 114 μm; Figure 2E) stage (Table 3). The GV oocytes from the VD3-treated follicles had larger (*P* < 0.05) diameters than those of the control follicles (Table 3).

**Table 3:** Characteristics of oocytes harvested from antral follicles at the end of culture.

	Number^a^ (n) of					Diameter (μm)	
Culture conditions	Follicles harvested	Oocytes harvested	Degenerate oocytes	Healthy	oocytes	GV oocytes	MI/II oocytes
				GV^b^	MI/II		
Control	17	17	6	11	0	91 ± 3	–
VD3	22	22	5	15	2	98 ± 2^∗^	108, 114

^a^*Data represent oocytes from 6 animals in each experimental group. Values are the mean ± SEM with each oocyte as an individual data point. ^∗^Significant difference with P < 0.05. ^*b*^VD3, 1α,25-dihydroxyvitamin D3 supplementation; GV, germinal vesicle; MI/II, metaphase I/II.*

## Discussion

Based on our previous data revealing positive impact of VD3 supplementation on follicular development, the current study further examined the direct actions of VD3 in the preantral and antral follicles. Moreover, experiments were conducted to investigate the expression of vitamin D biosynthesis enzymes and VDR in primate follicles developed *in vivo* and *in vitro*. Data suggest potential endocrine and paracrine/autocrine actions of vitamin D in the ovary, and a stage-specific trophic effect of vitamin D on follicular survival, growth and function, including oocyte maturation. For the first time, data suggest that follicular expression of vitamin D biosynthesis and signaling components appears to be influenced by VD3 levels present in the ovary.

Given that VDR is expressed in *in vivo*-developed follicles, VD3 originated from the circulation following biosynthesis in the liver and kidney could act directly on follicles to regulate their development. VDR was localized predominantly in the oocyte of follicles during the early developmental stages, especially the primordial and primary follicles. As follicle growth progressed, VDR was also evident in granulosa cells. Other than the current study, oocyte VDR expression has only been reported in fish (Bidman et al., 1997; Craig et al., 2008). In contrast, VDR expression in granulosa cells was noted in various species, including rodents, domestic animals, and humans (Johnson et al., 1996; Thill et al., 2009; Yao et al., 2017). The current data suggest the positive impact of VD3 on preantral follicle survival, as well as on both survival and growth of antral follicles developed *in vitro*. Recent research revealed that VD3 may exert its effect by altering reactive oxygen species production. VD3 addition decreased reactive oxygen species levels in cultured goat granulosa cells (Yao et al., 2017). An increase in plasma total antioxidant capacity was observed following vitamin D-K-calcium co-supplementation in vitamin D-deficient patients with polycystic ovary syndrome (Razavi et al., 2016). VD3 treatment was also reported to decreased caspase-3 activity in cultured human umbilical vein cell line (Ma et al., 2017). Thus, future studies need to further elucidate the role of vitamin D in maintaining and/or enhancing follicle health during early folliculogenesis, which could be through its antioxidant or anti-apoptotic actions, as well as by promoting granulosa cell proliferation and follicle growth during later stages of follicular development (Xu et al., 2016b; Yao et al., 2017). Approaches may include assessing expression of pro- and anti-apoptotic genes, as well as evaluating cell proliferation using bromodeoxyuridine as a marker (Ting et al., 2013).

The mRNA encoding vitamin D3 25-hydroxylase (*CYP2R1*) and 25-hydroxyvitamin D3-1α-hydroxylase (*CYP27B1*) are expressed in *in vivo*-developed follicles, which indicates that vitamin D3 biosynthesis could be achieved locally in the ovary. Although CYP2R1 and/or CYP27B1 mRNA or protein expression was reported in the human ovary (Bièche et al., 2007; Fischer et al., 2009) and macaque antral follicles (Xu et al., 2016c) (RNA-Seq of macaca mulatta: adult female ovarian follicle;^2^ accession number: SRP044327), neither was investigated in any species in terms of their regulation during the specific stages of follicular development. In addition to the endocrine actions of VD3, vitamin D3 and 25-hydroxyvitamin D3 in the circulation may serve as substrates for VD3 biosynthesis in the ovary, thereby supporting its paracrine and/or autocrine function in regulating follicular development. Moreover, the overall effects of vitamin D supplementation on ovarian function *in vivo* may depend on the form of vitamin D that is administered, i.e., cholecalciferol, calcifediol or calcitriol, as well as the expression and activity of vitamin D biosynthesis enzymes in the liver, kidney and ovary. Thus, studies are warranted to further investigate protein expression and cellular activities of vitamin D biosynthesis enzymes, as well as paracrine and autocrine function of vitamin D using follicle culture.

The current data demonstrate that *in vitro*-developed preantral and antral follicles express *VDR*, as well as *CYP2R1* and *CYP27B1*, which is similar to what is observed in *in vivo*-developed follicles. Notably, *VDR* expression decreased over the culture period from the preantral to the antral stage in the absence of VD3, i.e., under a vitamin D-depleted state according to media vitamin D assay performed previously (Xu et al., 2016b). Although having comparable sizes, *in vitro*-developed antral follicles expressed less *VDR* than *in vivo*-developed antral follicles. However, VD3 supplementation increased *VDR* expression in cultured antral follicles, particularly to the levels of *in vivo*-developed follicles. The data are consistent with previous studies, in which numbers of the DNA binding sites occupied by VDR were dynamically controlled by VD3 (Meyer et al., 2010; Ramagopalan et al., 2010). Although, VDR binding to its specific response elements was noted under vitamin D-depleted conditions, it was enhanced following VD3 administration, which was associated with increased VDR occupancy and elevated gene expression in mouse pre-osteoblastic cells (Meyer et al., 2010) and human lymphoblastoid cell lines (Ramagopalan et al., 2010). Thus, changes in follicular *VDR* expression during culture may correlate with numbers of accessible VDR binding sites in the absence or presence of VD3. Furthermore, follicular *CYP2R1* expression increased from the preantral to the antral stage under the control conditions, which was higher than *in vivo*-developed antral follicles. It could be that the loss of VD3 supply triggered the local biosynthesis in cultured follicles for active VD3 production. The elevation of *CYP2R1* expression in VD3-treated follicles may indicate the further demand of VD3, due to the increased VDR-mediated actions, that could not be met by the current VD3 supplementation at relatively low dose (Xu et al., 2016b). Therefore, to investigate vitamin D effects on ovarian function *in vivo*, multiple factors need to be considered, including patient initial conditions, as well as the dose and duration of vitamin D supplementation. Vitamin D-deficient patients may require initial vitamin D supplementation to prime adequate expression of VDR, as well as to balance the circulating vitamin D supply and local vitamin D biosynthesis, before significant effects could be revealed following additional vitamin D supplementation.

VD3 treatment at the current dose during the preantral stage did not alter follicular mRNA levels or media concentrations of *AMH*. In contrast, VD3 treatment during the antral stage increased media AMH concentrations, though not affecting *AMH* mRNA levels in *in vitro*-developed antral follicles. Because preantral follicle diameters were comparable between the control and VD3-treated groups, whereas antral follicle diameters were larger in VD3-treated follicles, the elevated media AMH concentrations may be due to the increased granulosa cell numbers in *in vitro*-developed antral follicles following VD3 treatment. Consistently, the relatively larger antral follicles following VD3 treatment produced higher levels of steroids in the media, e.g., E2, without altering mRNA levels of steroidogenic enzymes in the follicle, e.g., *CYP19A1*. Oocyte growth and maturation also correlated positively with antral follicle growth. Studies are warranted to investigate the expression of additional target genes that are activated by VD3 and may have potential roles in follicular development and oocyte maturation.

The numbers of macaques used are relatively small, which is a limitation of the current study. Research with increased sample sizes is needed to further elucidate vitamin D metabolism and signaling in the ovary. In conclusion, these data corroborate and extend previous findings in defining the significant impact of vitamin D and mechanisms of its actions upon folliculogenesis. This work demonstrates that vitamin D may regulate ovarian function by modulating expression of VDR and vitamin D intraovarian enzymes. Actions of vitamin D as an endocrine and paracrine/autocrine factor are significantly context-dependent in affecting follicular survival, growth and function. Factors that modulate intraovarian function of vitamin D *in vivo* could include the stage of follicular development, circulating baseline vitamin D status, and pharmacological levels of vitamin D exposure. These data may further underscore the concept that the underlying health status of a subject plays a role in influencing these dynamic and homeostatic intraovarian processes. Such baseline information may lead to a better understanding in the role of vitamin D in female reproduction, as well as provide insight into the potential mechanisms responsible for the reproductive benefits of vitamin D supplementation, such as in patients with polycystic ovary syndrome (Thys-Jacobs et al., 1999; Irani et al., 2014; Irani et al., 2015; Fang et al., 2017; Irani et al., 2017).

## Data Availability

All relevant data generated and analyzed for this study are included in the manuscript.

## Author Contributions

JX, DS, and JH contributed to the experimental design. JX, ML, FX, CB, DS, and JH contributed to the data analysis and interpretation. JX, ML, YD, and OT contributed to the follicle culture experiments. ML contributed to the histology experiments. JX, FX, CB, and LP-N contributed to the molecular biology experiments. JX, FX, DS, and JH contributed to the manuscript drafting. All authors contributed to manuscript revision, read and approved the submitted version.

## Conflict of Interest Statement

The authors declare that the research was conducted in the absence of any commercial or financial relationships that could be construed as a potential conflict of interest.

## Footnotes

1 http://www.ncbi.nlm.nih.gov/sra

2 http://www.ncbi.nlm.nih.gov/sra
